# Phosphatidic acid species 34:1 mediates expression of the myo-inositol 3-phosphate synthase gene *INO1* for lipid synthesis in yeast

**DOI:** 10.1016/j.jbc.2022.102148

**Published:** 2022-06-16

**Authors:** Maria Laura Gaspar, Manuel A. Aregullin, Yu-Fang Chang, Stephen A. Jesch, Susan A. Henry

**Affiliations:** Department of Molecular Biology and Genetics, Cornell University, Ithaca, New York, USA

**Keywords:** gene transcription, phospholipid metabolism, phosphatidic acid, phosphatidylinositol, fatty acids, DAG, 1,2-diacylglycerols, ER, endoplasmic reticulum, I, inositol, MRM, multiple reaction monitoring, PA, phosphatidic acid, PC, phosphatidylcholine, PE, phosphatidylethanolamine, PI, phosphatidylinositol, PS, phosphatidylserine, TAG, triacylglycerols, TCA, trichloroacetic acid, UAS_INO_, inositol-dependent upstream activating sequence

## Abstract

Depletion of exogenous inositol in yeast results in rising levels of phosphatidic acid (PA) and is correlated with increased expression of genes containing the inositol-dependent upstream activating sequence promoter element (UAS_INO_). *INO1*, encoding myo-inositol 3-phosphate synthase, is the most highly regulated of the inositol-dependent upstream activating sequence–containing genes, but its mechanism of regulation is not clear. In the current study, we determined the relative timing and kinetics of appearance of individual molecular species of PA following removal of exogenous inositol in actively growing wild type, *pah1*Δ, and *ole1*^*ts*^ strains. We report that the *pah1*Δ strain, lacking the PA phosphatase, exhibits a delay of about 60 min in comparison to wildtype before initiating derepression of *INO1* expression. The *ole1*^*ts*^ mutant on the other hand, defective in fatty acid desaturation, when grown at a semirestrictive temperature, exhibited reduced synthesis of PA species 34:1 and elevated synthesis of PA species 32:1. Importantly, we found these changes in the fatty acid composition in the PA pool of the *ole1*^*ts*^ strain were associated with reduced expression of *INO1*, indicating that synthesis of PA 34:1 is involved in optimal expression of *INO1* in the absence of inositol. Using deuterium-labeled glycerol in short-duration labeling assays, we found that changes associated with PA species 34:1 were uniquely correlated with increased expression of *INO1* in all three strains. These data indicate that the signal for activation of *INO1* transcription is not necessarily the overall level of PA but rather levels of a specific species of newly synthesized PA 34:1.

The anionic phospholipid phosphatidic acid (PA) is both a key precursor in lipid biosynthesis and a signaling molecule. In yeast, the level of PA in the endoplasmic reticulum (ER) plays a central role in the transcriptional regulation of lipid metabolism *via* the Opi1/Ino2-Ino4 (Henry) regulatory circuit (1, 2, 3, 4, 5, 6, 7, 8) (Fig. 1). PA is synthesized *de novo via* acylation of glycerol-3-P catalyzed by two fatty acyl CoA-dependent reactions as well as by turnover of individual phospholipids, *via* phospholipase D (9, 10). Cellular levels of PA are impacted by its rate of synthesis and by its rate of consumption as the central precursor to both phospholipids and the neutral lipids, diacylglycerol (DAG) and triacylglycerol (TAG) (4, 5, 9, 11). In growing cultures, PA is both rapidly synthesized and continuously converted into other lipid classes. Thus, despite its high rate of synthesis during active growth, the overall cellular levels of PA remain quite low amounting to about 1 to 3% of total cellular lipid content in wild type cells (12, 13). However, in cultures adapted to growth in synthetic medium in the absence of inositol, PA levels are significantly higher than those of cells grown in its presence (5, 13).

**Figure 1:**
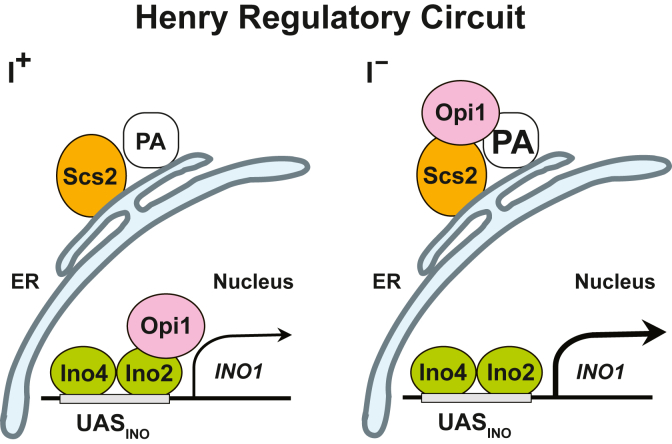
**Transcriptional regulation of the *INO1* gene *via* the Opi1/Ino2-Ino4 (Henry) regulatory circuit.** I^+^ (*left*) in wild type cells growing in the presence of inositol, transcription of the UAS_INO_-containing *INO1* gene is attenuated (*thin arrow*) by the interaction of Opi1with Ino2. I^−^ (*right*) upon inositol removal from the growth medium PI synthesis is reduced, leading to elevated PA levels and retention of Opi1 in the ER, thereby activating transcription of the *INO1* gene (*thick arrow*). UAS_INO,_ inositol-dependent upstream activating sequence.

The Opi1 repressor, a soluble transcription factor that binds to both PA and the integral membrane protein Scs2 in the perinuclear ER (1, 5, 6), plays a central role in transcriptional regulation of *INO1* and other inositol-dependent upstream activating sequence (UAS_INO_)-containing genes (1, 4, 14, 15, 16) (Fig. 1). In cells adapted to growth in the absence of inositol, phosphatidylinositol (PI) synthesis is dependent on *de novo* synthesis of inositol by the soluble enzyme myo-inositol 3-phosphate synthase encoded by the *INO1* gene (1, 4, 6, 17). Under these conditions, PI synthesis is reduced, and Opi1 remains associated with both PA and Scs2 in the ER allowing expression of *INO1* and other UAS_INO_-containing genes (Fig. 1). However, when inositol is abruptly added to cultures adapted to growth in the absence of inositol, PA levels drop precipitously as PI synthesis increases (1, 5). *In vitro* studies have revealed that Opi1 binds preferentially to PA over phosphatidylserine (PS) and to PA species in liposomes that contain saturated and shorter chain fatty acids (18). Moreover, the selective mechanism that enables binding of PA to Opi1 in preference to PS *in vitro* has been shown to involve a specific amphipathic helix spanning amino acids 109 to 138 (19).

While the interaction of Opi1 repressor with PA in the ER is vital to its cellular localization and to regulation of *INO1* and other UAS_INO_-containing genes (1, 5), the pool of PA in the ER consists of a range of PA species of diverse acyl-chain composition. Moreover, Pah1, a cytoplasmic Mg^2+^–dependent PA phosphatase that catalyzes the dephosphorylation of PA yielding DAG and P_i_ (20, 21), also plays a vital role in the relative synthesis of phospholipids *versus* DAG and TAG. Pah1 is recruited to the perinuclear ER when PA levels are elevated (22) through a process of phosphorylation and dephosphorylation. DAG generated by this reaction contributes primarily to the synthesis of TAG (21), as well as phosphatidylethanolamine and phosphatidylcholine (PC) *via* the Kennedy pathway (23, 24). However, the specific roles of individual PA species in transcriptional regulation of *INO1* and other UAS_INO-_containing genes have not been previously determined *in vivo*.

To gain insight into the potential roles of individual PA species in this complex metabolism and regulation process, we compared the overall kinetics of changes in levels of individual PA species with the kinetics of *INO1* derepression in both wild type and *pah1*Δ cells. We found that the kinetics of distribution of deuterium-labeled glycerol into PA species 34:1 in both strains was distinctly different from that observed in the overall cellular pool of PA. Moreover, PA species 34:1 was highly correlated with the kinetics of *INO1* expression in each of the two strains. More importantly, increased synthesis of PA species 34:1 was observed to precede the maximal induction of *INO1* expression in both wildtype and *pah1*Δ cells.

## Results

### The kinetics of *INO1* expression following removal of inositol in *pah1*Δ cultures differ from those in the wildtype strain

**Figure 2:**
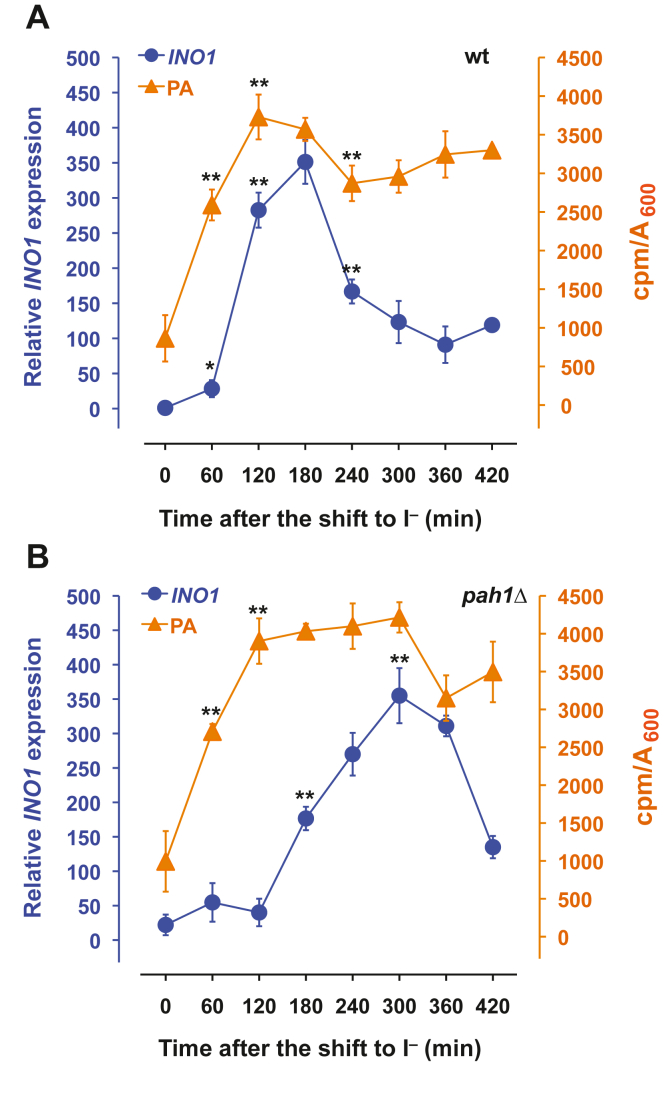
**Kinetics of *INO1* derepression and changes in PA composition following a shift to medium lacking inositol in wildtype and *pah1*Δ cells.** Cells were grown in I^+^ medium and phospholipids were labeled with 20 μCi/ml [^32^P]-orthophosphate to steady-state. At the mid-logarithmic phase of growth (A_600_ = 0.5), cells were filtered, resuspended in medium lacking inositol maintaining constant the specific activity of the label, and aliquots were taken at the indicated times. Data are expressed as counts of radiolabel ^32^P incorporated into total PA per OD units in the cell culture. For the analysis of *INO1* expression, cells were grown and harvested at the same time points as described above for the labeling of phospholipids. Total RNA was isolated and analyzed by RT-PCR as described in Experimental procedures. Panel *A* (wt); panel *B* (*pah1*Δ). Data are expressed as mean ± S.D. (n = 3), ∗∗*p* < 0.005 and ∗*p* < 0.05 *versus* time zero. PA, phosphatidic acid.

In wild type yeast cells, changes in the levels of PA occurring in the course of lipid metabolism during active growth provide a metabolic signal or signals regulating expression of many genes involved in phospholipid biosynthesis (1, 4, 5, 9). For purposes of the current investigation, the level of *INO1* expression in wild type cells growing at steady state in I^+^ medium was assigned a value of one unit (Fig. 2*A* time zero). As previously reported (5), following the shift of wildtype cells to I^−^ medium, both cellular PA content and *INO1* expression increased rapidly during the first 60 min. The most rapid increase in *INO1* expression occurred between 60 and 120 min following the shift to I^−^ medium, lagging by about 60 min behind the increase in PA levels (Fig. 2*A*). However, while PA levels remained elevated in wild type cells for the entire time course, *INO1* expression leveled off to about 100-fold over the fully repressed state, between 300 and 420 min in the absence of exogenous inositol, as cells adapted to *de novo* synthesis of inositol (Fig. 2*A*).

Cultures of *pah1*Δ exhibited about 20-fold higher expression of *INO1* than wild type grown under the same conditions (Fig. 2*B*). Moreover, after the shift to I^−^ medium, the kinetics of *INO1* derepression in *pah1*Δ cultures were strikingly different from that observed in wild type cells. Initially, *INO1* derepression in *pah1*Δ cells lagged by about 60 min in comparison to wild type under identical conditions (Fig. 2*B*). In addition, the level of *INO1* expression achieved in the *pah1*Δ strain by 180 min following the shift to I^−^ medium was only about half of that observed in wild type (Fig. 2, *A* and *B*). However, *INO1* expression in *pah1*Δ cells increased sharply after 120 min in I^−^ medium, reaching maximum expression at about 300 min (Fig. 2*B*). By 420 min in inositol-free medium, *INO1* expression, in both *pah1*Δ and wild type, had declined to similar levels of about 100- to 150-fold over fully repressed levels (compare Fig. 2, *A* and *B*).

### The shift of actively growing wild type cells from inositol containing to inositol free media induces dramatic changes in the fatty acid species composition of both membrane phospholipids and neutral lipids

**Figure 3:**
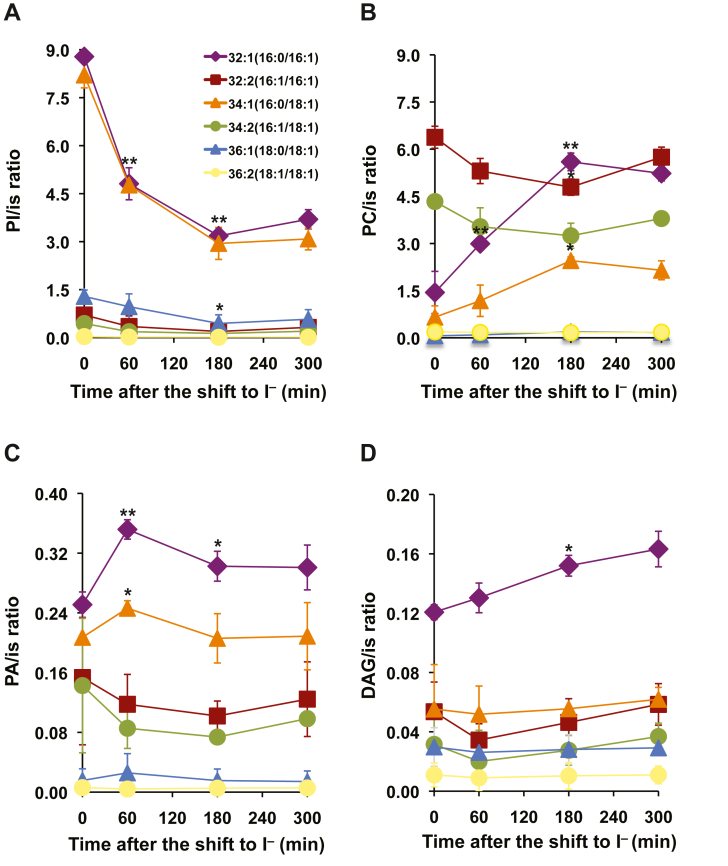
**Changes in lipid species profile in wildtype cells in response to inositol depletion.** Wild type cells were grown as described in Experimental procedures. Lipids were extracted, and the molecular species of lipids were analyzed by liquid chromatography coupled with electrospray ionization tandem mass spectrometry. The amount of the molecular species of each lipid was normalized to an internal standard (is). *A*, PI; *B*, PA; *C*, PC; *D*, DAG. The phospholipid species composition was as follows: 32:1 (*purple diamond*), 34:1 (*orange triangle*), 32:2 (*brown square*), C34:2 (*green circle*), 36:1 (*blue triangle*), 36:2 (*yellow circle*). Data are expressed as mean ± S.D. (n = 3), ∗∗*p* < 0.005 and ∗*p* < 0.05 *versus* wildtype time zero. DAG, 1,2-diacylglycerols; PA, phosphatidic acid; PC, phosphatidylcholine; PI, phosphatidylinositol; PS, phosphatidylserine.

Consistent with data obtained previously by labeling of total phospholipids to steady state with [^32^P]-orthophosphate in actively growing wildtype cultures (5, 11), mass spectrometry analysis revealed a decrease in the levels of all molecular species of PI following the shift of wild type cells to I^−^ medium (Fig. 3*A*). The major PI species, detected in wild type cells growing in I^+^ medium (Fig. 3*A*, time 0), were 32:1, 34:1, and 36:1. PI species 32:1 and 34:1 declined precipitously during the first 60 min after the shift to I^−^ medium in wild type cells (Fig. 3*A*), leveling off at about 180 min following the shift to I^−^ medium (Fig. 3*A*). Consequently, PA species 32:1 and 34:1 increased in the first 60 min following the shift to I^−^ medium (Fig. 3*C*) due to the decrease in the levels of PI species 32:1 and 34:1 in response to inositol depletion. In turn, PA species 32:1 and 34:1 declined after 60 min (Fig. 3*C*) to generate DAG species 32:1 and 34:1 (Fig. 3*D*). Subsequently, PC species 32:1 and 34:1 are derived from DAG species 32:1 and 34:1. These PC species increased during the first 180 min (Fig. 3*B*) mirroring the decline seen for PI species with the same acyl chain composition (Compare Fig. 3, *A* and *B*). Since the synthesis of PI is reduced in the absence of inositol, the increase in PC species 32:1 and 34:1 suggests that excess PA species 32:1 and 34:1, normally used for PI biosynthesis, are being diverted into increased PC synthesis.

**Figure 4:**
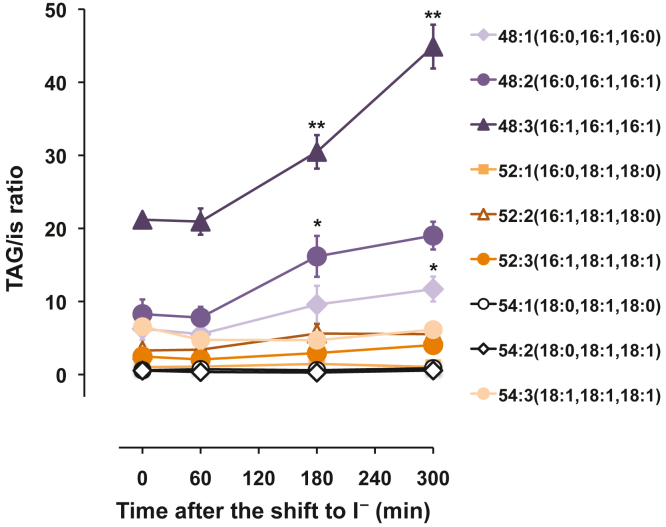
**Inositol depletion influences the composition of the TAG molecular species in wildtype cells.** Wild type cells were grown as described in Experimental procedures. Lipids were extracted and the molecular species of lipids were analyzed by LC-ESI-MS/MS. The amount of the molecular species of TAG was normalized to an internal standard (is). Data are expressed as mean ± S.D. (n = 3), ∗∗*p* < 0.005 and ∗*p* < 0.05 *versus* wildtype time zero. LC-ESI-MS/MS, liquid chromatography coupled with electrospray ionization tandem mass spectrometry; TAG, triacylglycerol.

As expected, TAG species were also impacted by the shift to I^−^ medium. The most abundant TAG species in cells grown in I^+^ medium (Fig. 4) were TAG species 48:3 (containing three C16:1 acyl chains) followed by TAG species 48:2 (containing one C16:0 acyl chain and two C16:1 acyl chains) and TAG species 48:1 (containing two C16:0 acyl chains and one C16:1 acyl chain) (Fig. 4). Following the shift to I^−^ medium, levels of TAG species 48:3 dramatically increased from 60 to 300 min (Fig. 4). TAG species 48:2 and 48:1 also significantly increased but to a lesser extent (Fig. 4). More importantly, evidence presented here indicates that TAG is accumulating in wild type cells upon inositol depletion. As shown above, the absence of inositol in the media altered PI metabolism significantly resulting in increased levels of PA. Under these circumstances, PA is in part a reservoir of fatty acids for the synthesis of TAG. Together, these results suggest that synthesis of storage lipids and membrane-forming lipids are interdependent and operate in a coordinated fashion to maintain lipid homeostasis in yeast cells.

### Differential synthesis of PA species in response to inositol depletion in wild type and *pah1*Δ

**Figure 5:**
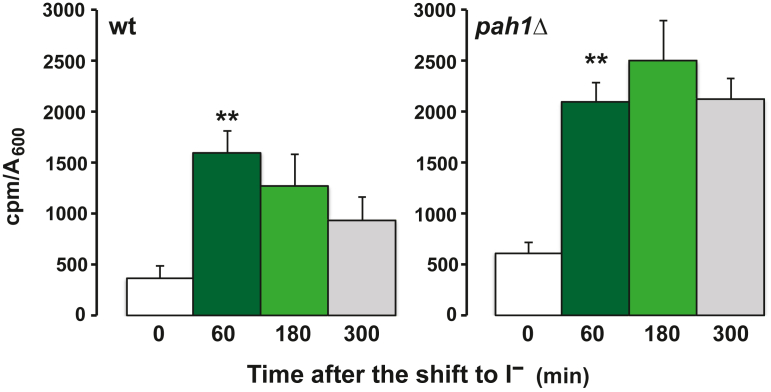
**PA synthesis is affected by removal of inositol from the medium in wildtype cells and *pah1*Δ.** Cells were pregrown in I^+^ medium until mid-logarithmic phase of growth (A_600_ = 0.5). At this time point, cells were filtered, washed with prewarmed medium lacking inositol, and resuspended in I^−^ medium at 30 °C. 100 μCi/ml [^32^P]-orthophosphate was immediately added to the cultures shifted to I^−^ medium, and these cells were harvested 25 min later. This sample is referred to as the 0 time-point. ^32^P was added in similar fashion to cultures at 60, 180, and 300 min following the media shift. For each time point, samples were harvested 25 min after addition of label. Lipids were extracted and analyzed as described in Experimental procedures. Data are expressed as counts of radiolabel ^32^P incorporated into total PA per OD units in the cell culture. Data are expressed as mean ± S.D. (n = 3) and ∗∗*p* < 0.005 *versus* time zero. PA, phosphatidic acid.

Since PA is precursor to both membrane forming phospholipids and the neutral storage lipid, TAG, *via* DAG, we initially hypothesized that the decline in PA levels in wild type cells between 120 and 240 min of growth in I^−^ medium (Fig. 2*A*) might be due to increased consumption of DAG, as precursor to the synthesis of TAG (Fig. 4). However, DAG also serves as a precursor to PC synthesis (Fig. 3*B*). To test this idea, we used the *dga1*Δ *lro1*Δ *are1*Δ *are2*Δ strain, defective in synthesis of the neutral lipids DAG and TAG, and a quintuple mutant strain (*dga1*Δ *lro1*Δ *are1*Δ *are2*Δ *pct1*Δ), lacking the ability to synthesize TAG and PC through the CDP-choline pathway. Thus, if the hypothesis that preventing the flow of PA into the synthesis of TAG and PC was correct, the collective mutations in the quintuple mutant should have resulted in higher *INO1* expression following the shift to I^−^ medium. However, contrary to this hypothesis, we found that the quintuple mutant exhibited kinetics of *INO1* derepression and attenuation quite similar to those observed in wildtype cells. These results suggest that the pattern of *INO1* derepression and attenuation in wild type cells, during the time course following the shift to I^−^ medium (Fig. 2*A*), does not respond to the pool of PA used to synthesize TAG and PC *via* the CDP-choline pathway. Instead, the distinctive kinetics of *INO1* derepression between wild type and *pah1*Δ may be responding to the rate of PA synthesis and its short-term retention prior its utilization as a precursor in the synthesis of other lipids. To assess the relative rate of PA synthesis in the wild type and *pah1*Δ strains, cells were maintained in logarithmic phase and pulse-labeled with [^32^P] orthophosphate for 25 min at 0, 60, 180, and 300 min, following the shift to I^−^ medium (Fig. 5). Strikingly, under these conditions, wild type cells incorporated and/or retained significantly less ^32^P in PA than that observed in the *pah1*Δ strain, especially at 180 min (Fig. 5). Within 60 min following the shift to I^−^ medium, ^32^P label associated with PA increased in both strains by about 4-fold. As expected, the level of ^32^P associated with PA in wild type declined steadily from 60 to 300 min after the shift (Fig. 5). However, due to the absence of the Pah1 phosphatase, the level of ^32^P associated with PA in *pah1*Δ remained proportionally higher than in wild type in each of the time pulses (Fig. 5). Also, consistent with the loss of the Pah1 PA phosphatase, ^32^P associated with PA in the *pah1*Δ strain was initially higher even at 0 min after the shift, in contrast to wild type, and failed to decline in the interval between 60 and 300 min after the shift to I^−^ medium (Fig. 5). Overall, however, these data indicate that the increase in the proportion of label incorporated into PA in both wild type and *pah1*Δ strains preceded the maximal induction of *INO1* (Compare Figs. 2, *A* and *B* and 5).

The substantial increase in the amount of ^32^P label associated with PA between 0 and 60 min in wild type cells following the shift to I^−^ medium (Fig. 5), suggested that the increase in PA synthesis may reflect the increase of PA species 32:1 and 34:1 observed in Figure 3*B*. To investigate the relative rates of synthesis of individual PA species over a period of 300 min correlated with *INO1* expression, we used deuterium-labeled glycerol to perform short time labeling experiments at various time points following a shift to I^−^ medium.

**Figure 6:**
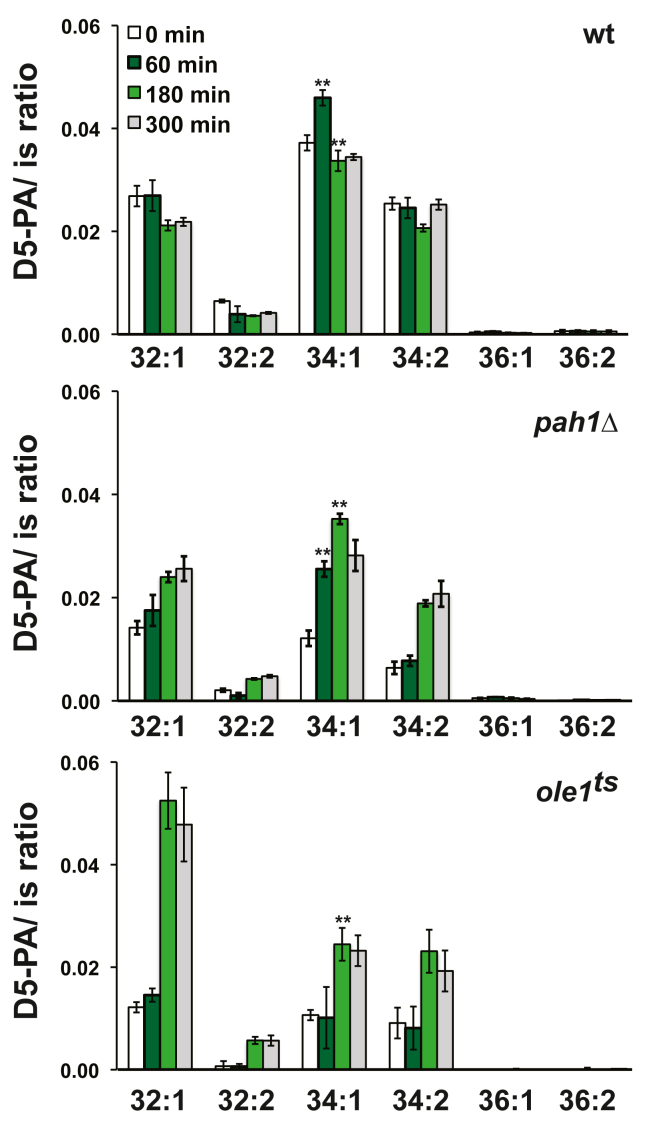
**Differential synthesis/retention of newly formed PA species in response to inositol depletion.** Wild type, *pah1*Δ, and *ole1*^*ts*^ cells pregrown in I^+^ medium until mid-logarithmic phase of growth (A_600_ = 0.5) at 30 °C. At this time point, cells were filtered, washed with prewarmed medium lacking inositol, and resuspended in I^−^ medium at 30 °C. Deuterium-labeled glycerol was immediately added to the cultures shifted to I^−^ medium, and these cells were harvested 25 min later. This sample is referred to as the 0-time point. Deuterium-labeled glycerol was added in similar fashion to cultures at 60, 180, and 300 min following the media shift. For each time point, samples were harvested 25 min after addition of label. Lipids were extracted and analyzed by LC-ESI-MS/MS as described in Experimental procedures. The amount of lipids was normalized to an internal standard (is). The data are the averages of two separate experiments ±SD and ∗∗*p* < 0.005 *versus* time zero. D5-PA indicates deuterium labeled phosphatidic acid. LC-ESI-MS/MS, liquid chromatography coupled with electrospray ionization tandem mass spectrometry; PA, phosphatidic acid.

As shown in Figure 6, both, wild type and *pah1*Δ cells incorporated deuterium-labeled glycerol primarily into PA species 32:1, 34:1, and 34:2. However, in contrast to wild type, a continuous increase in label associated with PA species 32:1 and 34:2 was observed in *pah1*Δ cells (Fig. 6), suggesting that failure to dephosphorylate these specific PA species might play a role in the altered kinetics of *INO1* expression in the *pah1*Δ strain *versus* wild type (Fig. 2). In wild type cells, incorporation of label into these PA species was similar at 0 and 60 min after the media shift, followed by a decrease in label incorporated into PA species 32:1 and 34:2 at 180 min (Fig. 6). Strikingly, the label associated with PA species 34:1 increased significantly between 0 and 60 min following the shift to I^−^ medium in both wild type and *pah1*Δ strains (Fig. 6). Moreover, the timing of the relative increase in deuterium label incorporated into PA species 34:1 correlates with the kinetics of *INO1* expression in both wild type and *pah1*Δ following the shift to I^−^ medium (Compare Figs. 2, *A* and *B* and 6).

### Changes in the synthesis of PA species 32:1 and 34:1 after removal of inositol in the *ole1*^*ts*^ mutant relative to wildtype are correlated to inability to derepress *INO1*

**Figure 7:**
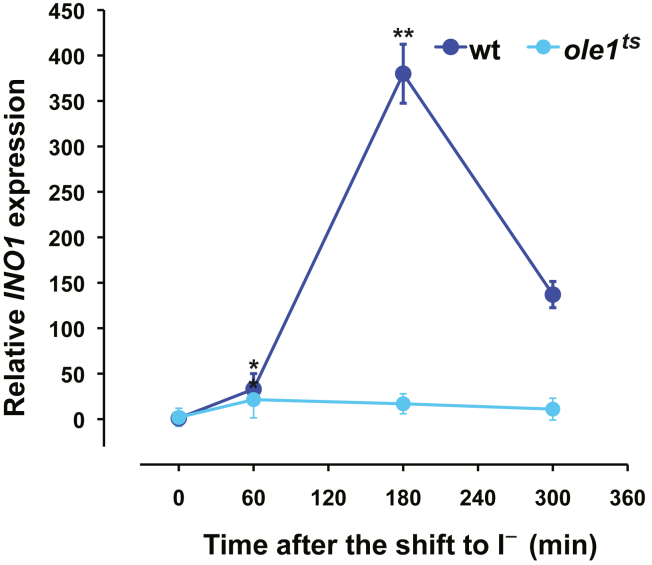
**Effect of inositol depletion on the expression profile of *INO1* in the *ole1***^***ts***^**strain.** For the analysis of *INO1* expression, cells were grown on medium containing inositol to mid-logarithmic phase at 30 °C. Cultures were filtered, washed with prewarmed medium lacking inositol, and resuspended in new medium lacking inositol, maintaining the same temperature. Samples were harvested by filtration at the indicated time points, flash-frozen in dry ice, and stored at −80 °C. Total RNA was isolated and analyzed by RT-PCR as described in Experimental procedures. Data are expressed as mean ± S.D. (n = 3), ∗∗*p* < 0.005 and ∗*p* < 0.05 *versus* time zero.

The increase in the synthesis/retention of the PA species 34:1 observed in both wild type and *pah1*Δ (Fig. 6) between 0 and 60 min is correlated with the burst in *INO1* expression that occurs after 60 min following removal of inositol (Fig. 2). PA species 34:1 contains either one C16:0 acyl chain and one C18:1 acyl chain or one C16:1 acyl chain and one C18:0 acyl chain. As Ole1 is the sole fatty acid desaturase in yeast (25), it is responsible for generating both the 16:1 and 18:1 unsaturated fatty acids. Thus, we reasoned that reduced production of these unsaturated fatty acids in the *ole1*^*ts*^ strain should result in diminished synthesis of the PA species 34:1 and, in turn, influence the kinetics of derepression of the *INO1* gene. To test this hypothesis, we used a temperature sensitive strain, *ole1*^*ts*^ (26) to examine and compare the kinetics of synthesis of both PA species relative to the kinetics of increasing *INO1* expression following the shift to I^−^ medium. However, the growth of the *ole1*^*ts*^ mutant at 37 °C is significantly reduced even in medium containing inositol, and cells of this strain died shortly after the shift to I^−^ medium (data not shown). For this reason, experiments were carried out at the semipermissive temperature of 30 °C, while maintaining cultures in logarithmic phase. Cells were labeled with deuterium-labeled glycerol for 25 min at the same time points as described above for wild type and *pah1*Δ. At time 0, the label incorporated into PA species 32:1 in the *ole1*^*ts*^ strain was half the value observed in wild type cultures under identical conditions (Fig. 6). The PA species that showed the greatest increase in labeling in ole1^ts^ under these circumstances was PA 32:1 (Fig. 6). This observation suggests that the *ole1*^*ts*^ mutant can increase synthesis of 16:1 and 18:1 if incubated for a longer period in I^−^ medium at the semipermissive temperature of 30 °C. Under these circumstances, the label associated with PA species 34:1 was 4-fold lower than the value detected in wildtype (Fig. 6). The amount of label associated with PA species 32:1, 34:1, and 34:2 in *ole1*^*ts*^ at 60 min after the media shift was like the levels found at zero time (Fig. 6). The increase in the label incorporated into these PA species at later time points (Fig. 6) could be due to breakdown of storage lipids, which can supply the demands for unsaturated fatty acids for a short period of time. Importantly however, *INO1* was not expressed to any significant extent in the *ole1*^*ts*^ mutant after the shift to I^−^ medium and increased by only 10-fold overall as compared to 350- to 400-fold in wild type (Fig. 7).

## Discussion

Addition of inositol to actively dividing wild type cells adapted to growth in I^−^ medium triggers a rapid decline in PA levels, providing the signal for repression of *INO1* (1, 4, 5) (Fig. 1). Indeed, *INO1* expression declines by more than 400-fold within about 30 min following the addition of inositol (1, 4, 5). However, such rapid and almost simultaneous changes in both metabolism and gene expression can obscure the detection of transient metabolic signals. In contrast, shifting wildtype cells adapted to growth in I^+^ medium to I^−^ medium results in derepression of *INO1* and other UAS_INO_-containing genes over a period of hours, as opposed to minutes, as cellular stores of inositol are gradually depleted due to ongoing metabolism (5). In the current study, we exploited the slower patterns of changes in both lipid metabolism and gene expression to gain insight into the potential roles of individual PA species in this complex metabolism and regulation. Following the shift of both wild type and *pah1*Δ cells to I^−^ medium, overall PA levels rose with similar kinetics in the two strains (Fig. 2). However, derepression of *INO1* was delayed in *pah1*Δ cells in comparison to wild type. This finding indicates that the activity of the Pah1 PA phosphatase is instrumental in controlling a pool of PA critical for the rapid derepression of *INO1* in wild type cells following a shift to I^−^ medium.

In addition, in the course of ongoing metabolism, there are multiple sources that generate and utilize PA, thus having the potential to affect the expression of UAS_INO_-containing genes, especially *INO1*. PA is the central precursor in the synthesis of both membrane phospholipids and the major storage lipid TAG. In growing cells, PA is generated in an ongoing fashion by *de novo* from glycerol-3-P and acyl-CoA, but also through Dgk1 DAG kinase activity, which catalyzes the phosphorylation of DAG to PA (27, 28). Phospholipase D-mediated turnover of PC also results in the production of PA and free choline (9). While phospholipase D activity is localized mainly in the plasma membrane and endosomes (29, 30), the enzymes that catalyze the *de novo* synthesis of PA are found in the ER and lipid droplets (31, 32). We favor the idea that *INO1* derepression in the absence of inositol is responding to the pool of PA that is newly synthesized in the ER. Our reasoning is based in a previous study in which we demonstrated that upon addition of inositol into the growth medium, newly synthesized PA is rapidly depleted in the ER in direct response to a rapid increase in PI synthesis (1). Our current analysis using deuterium-labeled glycerol is consistent with the hypothesis that a specific pool of newly synthesized PA precedes the burst in *INO1* expression following inositol depletion. We observed that in wildtype cells, deuterium-labeled glycerol was incorporated preferentially into PA species 34:1 followed by PA species 32:1, 34:2 and 32:2 (Fig. 6). However, PA species 34:1 was the only molecular species of PA that exhibited an increase in the rate of synthesis from 0 to 60 min (Fig. 6), suggesting that the burst in *INO1* expression that occurs from 60 to 120 min after the shift to I^−^ medium may be responding to the abundance of this PA molecular species, as opposed to the overall cellular pool of PA. Incidentally, this molecular species is the major one found in wild type and *pah1*∆ cells (33). Importantly, the increase in the synthesis of the PA species 34:1 precedes the spike in INO1 expression, and this PA species must reach certain threshold to induce expression of *INO1*, which it is met or exceeded in the case of wildtype and *pah1Δ* but not in *ole1*^*ts*^ mutant.

Previous studies have characterized the PA species profile of wild type and *pah1*Δ grown in YEPD medium (33) and in wildtype cells grown in medium containing different carbon sources (34). Indeed, the composition of the growth medium has a profound influence on the PA species profile, which exhibit a remarkably degree of variability (34). In the current study, we report the changes in the levels of PA species in wild type following a shift to medium lacking inositol (Fig. 3*C*). Since PA is the entry point to the phospholipid biosynthetic pathway, the relative amounts of the different PA molecular species depend on their utilization in the generation of lipids such as PI and PC (Fig. 3, *A* and *C*). It is interesting to note that once an acyl chain has been transferred from acyl-CoA to a PA backbone, it is no longer susceptible to elongation or desaturation (35). Consequently, PA can be seen as a moderator between the free acyl-CoA pool and the acyl-CoA bound to phospholipids.

The Opi1 repressor controls the expression of *INO1* *via* the Opi1/Ino2-Ino4 (Henry) regulatory circuit (1, 2, 3, 4, 5, 6, 7, 8) (Fig. 1). In the absence of inositol, Opi1 interacts with PA in the perinuclear ER, Opi1 contains a motif, FFAT (two phenylalanines in an acid tract), shown by Loewen *et al.* (2003) (36) to interact with an integral ER-associated protein Scs2 (Fig. 1). Since Opi1 tethering to the ER requires interaction with both Scs2 and PA, Opi1 remains in the ER when cells are growing in the absence of inositol, resulting in derepression of *INO1* and coregulated UAS_INO_-containing genes (1) (Fig. 1). Both the headgroup and the acyl chains of PA are critical in the binding of Opi1 to the ER membrane. Kooijman and Burger (37) pointed out that unsaturated PA has a special cone shape that facilitates its interaction with proteins. To our knowledge, the present study has revealed for the first time that the PA species 34:1 may be involved in the recruitment of Opi1 to the ER in response to a shift to medium lacking inositol. However, *in vitro* liposome binding assays have shown that the Opi1 repressor exhibited higher binding affinity toward shorter-chain and more-saturated PA species (18). However, the relative specific affinity of the Opi1 repressor for the PA molecular species 34:1 has yet not been tested and compared to other PA species. Moreover, liposome binding assays lack the influence of Scs2, which is required for the interaction of Opi1 to PA *in vivo* (1).

In summary, we have found that the kinetics of *INO1* derepression in wild type cultures that have been shifted to medium depleted of inositol are influenced not only by the overall level of PA but also by the rate of PA synthesis. The synthesis of the PA species 34:1 appears to be critical for the induction of *INO1* in the absence of inositol. Further studies are necessary to determine if this PA species interacts *in vivo* with the Opi1 repressor.

## Experimental procedures

### Culture conditions

Cultures were maintained on YEPD (1% yeast extract, 2% peptone, 2% glucose, 2% agar) media plates. All experiments were conducted using cultures grown to mid-logarithmic phase at 30 °C on a rotary shaker (New Brunswick Scientific Co, Inc) at 200 rpm using chemically defined synthetic media as described by Jesch *et al.* (38). Cells were grown in 50 ml batches of complete synthetic media with (I^+^) or without (I^−^) inositol (75 μM) as indicated. Solid media had the same composition plus 2% agar.

### Strains

Yeast strains used are listed in Table 1. Deletion mutant strains were generated by PCR-mediated gene replacement as described previously (40). The plasmid pRS315 was used as a template to generate a PCR fragment for the *PAH1* and *PCT1* gene disruption. The entire open reading frame of the *PAH1* and *PCT1* genes were replaced with the *LEU2* marker gene. Leucine prototrophs were screened by colony PCR to verify integration at the correct genetic locus.

**Table 1:** List of yeast strains used in this study

Strain	Genotype	Source or ref
BY4742	MATα *his3*Δ *leu2*Δ*0 lys2*Δ*0 ura3*Δ*0*	Invitrogen
LGY371	MATα *pah1*Δ*::LEU2 his3*Δ *leu2*Δ*0 lys2*Δ*0 ura3*Δ*0*	This study
YJP1078	MATα *lro1Δ::Kan*MX *dga1Δ::Kan*MX *are1Δ::Kan*MX, *are2Δ::Kan*MX *his3*Δ*1 leu2*Δ*0 lys2*Δ*0 ura3*Δ*0*	(39)
LGY336	MATα *lro1Δ::Kan*MX *dga1Δ::Kan*MX *are1Δ::Kan*MX *are2Δ::Kan*MX *pct1Δ::LEU2 his3*Δ*1 leu2Δ0 lys2*Δ*0* *ura3*Δ*0*	This study
LGY164	MATα *pct1*Δ*::LEU2 his3*Δ *leu2*Δ*0 lys2*Δ*0 ura3*Δ*0*	This study
SJY484	MATα *his7*Δ *lys2*Δ *ura1Δ tyr1Δ gal1Δ ade1Δ ade2Δ*	(26)
SJY485	MATα *ole1 his*7Δ *lys2*Δ *ura1Δ tyr1Δ gal1Δ ade1Δ ade2Δ*	(26)

### Materials

All chemicals were reagent grade. Phospholipid standards were purchased from Avanti Polar Lipids. Neutral lipid standards were purchased from Nu-Chek Prep. Deuterium-labeled glycerol (1,1,2,3,3-D5, 99%) was purchased from Cambridge Isotope Laboratories, Inc. [^32^P]-orthophosphate was purchased from PerkinElmer, and scintillation-counting supplies were purchased from National Diagnostics. Silica gel loaded SG81 chromatography paper was purchased from Whatman, Inc, and HPTLC plates from Merck. Growth medium supplies were purchased from Difco Laboratories.

### RNA isolation and RT-PCR analysis

To measure expression of the *INO1* gene, cells were grown on medium containing inositol to mid-logarithmic phase at 30 °C and allowed to grow to A_600_ = 0.5 to 0.6. Cultures were filtered, washed with prewarmed medium lacking inositol, and resuspended in new medium lacking inositol, maintaining the same temperature. Samples were harvested by filtration at every hour for a period of 5 to 7 h following the media shift, flash-frozen in dry ice, and stored at −80 °C.

Total RNA was isolated using RNeasy Mini Kit including a DNA digestion with RNase-free DNase Set (both Qiagen). RNA (1 μg) was transcribed into cDNA using oligo(dT) 12-18 primer (0.5 μg), PCR-grade dNTP mix (0.5 μM), first strand buffer (1×), DTT (10 mM), and 100 Units SuperScript III Reverse Transcriptase (Invitrogen). Real-time PCR was performed on a StepOnePlus Real-Time PCR System (Applied Biosystems) using TaqMan Universal PCR Master Mix, No AmpErase UNG (Applied Biosystems) and the following TaqMan probes and primers: *INO1*: TaqMan probe, 5′FamCTG TTG CCC ATG GTT AGC CCA AAC G-Tamra3’; forward primer, 5′GGA ATG ACG TTT ATG CTC CTT TTA A3’; reverse primer, 5′GTC CCA ACC AGA GAC GAC AAA3’; *ACT1*: TaqMan probe, 5′FamTGC AAA CCG CTG CTC AAT CTT CTT CAA T-Tamra3’; forward primer 5′CGC CTT GGA CTT CGA ACA AG3′, reverse primer, 5′GAC CAT CTG GAA GTT CGT AGG ATT3’. *ACT1* gene served as an internal standard for normalization.

In brief, the reaction mix in a volume of 25 μl consisted of 0.5 μM primers, 0.2 μM TaqMan probe, 1× master mix, and 5 ng cDNA. All reactions were performed in technical duplicate. Nontemplate control (5 ng RNA) and nonreaction control (DEPC-water) were routinely performed. The thermal program for the PCR included stage 1: 95 °C, 10 min, stage 2: 95 °C, 0.5 min, and 60 °C, 1 min for a total of 40 cycles, and stage 3: hold at 4 °C. Relative quantitation was done using the ΔΔC*t* method (see StepOnePlus user manual of Applied Biosystems). The ΔΔC*t* represents the change in mRNA expression after *ACT1* normalization relative to the wildtype control calculated as 2^−(*Gene*^ ^C*t*^_x_^−^^*ACT1*^ ^C*t*^_x_^) − (*Gene*^ ^C*t*^_cr_^−^^*ACT1*^ ^C*t*^_cr_^)^, where “gene” represents the mRNA under study (*INO1*), “x” refers to the strain from which the mRNA to be tested was derived (*i.e.*, wildtype or quadruple mutant), and “cr” refers to the control mRNA. The value “cr” (for control mRNA) was derived from level of mRNA in the wildtype strain, pregrown as described above in I^+^ medium at 30 °C, but shifted to fresh medium of the same composition (*i.e.*, containing inositol) at the same temperature. The “C*t*” (cycle threshold) is defined as the number of cycles required for the fluorescent signal to cross the threshold (*i.e.*, to exceed background level). Each RT-PCR experiment was performed at least in triplicate.

### Kinetic analysis of changes in PA composition following inositol addition

To determine changes in the composition of PA over a set time course following inositol depletion, cells were grown in I^+^ medium, and phospholipids were labeled with 20 μCi/ml [^32^P]-orthophosphate (specific activity of isotope was 2.7 mCi/mmole phosphate) to steady-state. When the cells reached the mid-logarithmic phase of growth (A_600_ = 0.5) were filtered, washed with prewarmed medium lacking inositol, and resuspended in I^−^ medium maintaining constant the specific activity of the label. Cultures were incubated at 30 °C for additional 300 min. Five milliliters samples were taken at 0, 60, 120, 180, 240, and 300 min and were mixed with 0.5 ml 50% trichloroacetic acid (TCA) and allowed to stand on ice for 20 min. The samples were washed twice with distilled water. Labeled lipids were extracted as previously described (41). The individual phospholipids species were resolved by two-dimensional paper chromatography (42). Phospholipid identity was based on the mobility of known standards and quantified on a STORM 860 PhosphorImager (Amersham Biosciences).

### Analysis of PA synthesis following inositol deprivation

To determine changes in the synthesis of PA over a set time course following inositol starvation, cells were pregrown in I^+^ medium until mid-logarithmic phase of growth (A_600_ = 0.5). At this time point, cells were filtered, washed with prewarmed medium lacking inositol and resuspended in I^−^ medium at 30 °C. 100 μCi/ml [^32^P]-orthophosphate (specific activity of isotope was 13.51 mCi/mmole phosphate) was immediately added to the cultures shifted to I^−^ medium, and these cells were harvested 20 min later. This sample is referred to as the 0-time point. ^32^P was added in similar fashion to cultures at 60, 180, and 300 min following the media shift. For each time point, samples were harvested 20 min after addition of label. Five milliliters samples were mixed with 0.5 ml 50% TCA and allowed to stand on ice for 20 min. The samples were washed twice with distilled water. Lipids were extracted and analyzed as described above for ^32^P steady-state labeling.

### Electrospray ionization mass spectrometry analysis of lipids

To analyze the phospholipid and neutral lipid species following a shift to medium lacking inositol, cells were pregrown in I^+^ medium until mid-logarithmic phase of growth (A_600_ = 0.5). The cells were then shifted as described above to medium lacking inositol and incubated for additional 300 min. Two sets of samples (A_600_ = 25), one set to be used for phospholipid extraction and the other set to be used for neutral lipid extraction, were collected at 0, 60, 180, and 300 min by rapid filtration through Durapore membrane filters (0.65 μm DVPP. Millipore, Inc) and immediately transferred to a tube containing 5 ml of 10% TCA in an ice bath for 25 min. The cell membranes were spun down, and the supernatant discarded. The pellet was washed twice with water. The cell membrane phospholipids were extracted with 1 ml of a solvent mixture consisting of ethanol, water, ethyl ether, pyridine, and ammonium hydroxide (45:45:15:3:0.003, v/v/v/v/v). Fifty microliter of 0.05 mg/ml phospholipid internal standards (PA 28:0, PC 34:0 and PI from bovine liver) were added to the extraction mixture. Samples were sonicated for 20 min and incubated at 60 °C for 50 min. Cell debris were pelleted by centrifugation at 5000*g* for 10 min, and the supernatant was collected. The supernatant was dried under a stream of nitrogen. The lipid film was resuspended in 300 μl of chloroform: methanol 1:1 v/v for MS analysis.

The cell neutral lipids were extracted with a mixture of chloroform: methanol 2:1 v/v. 50 μl of 0.05 mg/ml neutral lipid internal standards (DAG 28:0 and TAG 51:0) were added to the extraction mixture. Samples were sonicated for 20 min and incubated at room temperature for 60 min. The chloroform phase was dried, and the residue was dissolved in 300 μl of chloroform: methanol 1:1 v/v for MS analysis.

Quantitative analysis of polar and neutral lipid species was performed using a Dionex UltiMate 3000 HPLC system (Thermo Fisher Scientific) configured for microbore and outfitted with fused silica transfer lines coupled with an AB/Sciex 4000 Q Trap mass spectrometer (Applied Biosystems) fitted with a Turbo Ion Spray source operated in multiple reaction monitoring (MRM) mode. In MRM experiments, it was provided a unit resolution for both Q1 and Q3, 50 ms dwell time for each transition ion pair and Q1 and Q3 MRM transition pairs. The standards PA 28:0, PC 34:0, DAG 28:0, TAG 51:0, and PI from bovine liver were diluted to 20 ng/μl and infused at 5 μl/min directly into the mass spectrometer and relevant MRM parameters optimized using parameter ramping following manufacturer’s recommended protocol. PA and PI lipid species were optimized for acquisition in negative ion mode as deprotonated adducts [M − H]^−^, PC lipid species were optimized for acquisition in positive ion mode as protonated adducts [M + H]^+^, DAG, and TAG lipid species were optimized for acquisition in positive ion mode as lithium adducts [M + Li]^+^. Samples for PA and PI species analysis were diluted 1:20 with eluent A in glass insert autosampler vials (SUN-SRi) with an injection volume of 5 μl using a user defined program with eluent A in pick-up vials 1 and 2 and infused at a rate of 65 μl/min and separated on a Viva Silica 5μ, 300A, 200 × 1 mm (Restek) column at 30 °C. The separation was carried out using a linear gradient as follows: 0-2-20-24-25-38 min, 10-10-100-100-10-10 % eluent B, mass spectrometer data acquisition start trigger at 2 min [Eluent A: 58/40/2 isopropanol/hexane/water (v/v/v) with 0.2% glacial acetic acid (v/v) and 50 mM ammonium acetate (w/v); eluent B: 50/40/10 isopropanol/hexane/water (v/v/v)] with 0.2% glacial acetic acid (v/v) and 50 mM ammonium acetate (w/v)]. Samples for PC species analysis were treated as above and analyzed using a slightly different linear gradient as follows: 0-1-32-35-36-48 min, 5-5-85-85-5-5 % eluent B, mass spectrometer data acquisition start trigger at 2 min and an injection volume of 2 μl using user defined program with eluent A in pick-up vials 1 and 2. The samples for DAG and TAG species analysis were diluted 1:10 (v/v) with eluent A in glass insert autosampler vials with an injection volume of 10 μl using user defined program with eluent A in pick-up vials 1 and 2 and infused at a rate of 60 μl/min and separated on an Ultra C1 3, 100A, 150 × 1 mm (Restek. Bellefonte, PA) column at 30 °C. The separation was carried out using a linear gradient as follows: 01.5-2-30-31-36-37-52 min, 0-0-5-75-100-100-0-0 % eluent B, mass spectrometer data acquisition start trigger at 1 min [Eluent A: 60/20/20 methanol/isopropanol/water (v/v/v) with 0.2% glacial acetic acid (v/v), 50 mM ammonium acetate (w/v), and 2 mM lithium acetate (w/v); Eluent B: 5/90/5 methanol/isopropanol/water (v/v/v) with 0.2% glacial acetic acid (v/v), 50 mM ammonium acetate (w/v), and 2 mM lithium acetate (w/v)]. Raw data were acquired using Analyst, version 1.6.1 (AB/Sciex). and raw MRM data were processed using MultiQuant, version 2.1.1 (AB/Sciex).

### Short-time labeling with deuterium-labeled glycerol

Cells grown to mid-logarithmic phase in I^+^ medium were collected by filtration, washed with prewarmed medium lacking inositol, and resuspended in I^−^ medium at 30 °C. A 25 ml sample was immediately transferred to a different flask and supplemented with 50 mM deuterium-labeled glycerol (1,1,2,3,3-D5, 99%). These cells were harvested 25 min later. This sample is referred to as the zero-time point. Deuterium-labeled glycerol was added in similar fashion to cultures at 60, 180, and 300 min after the shift to I^−^ medium. For each time point, 25 ml samples were harvested by filtration 25 min after addition of the deuterium-labeled glycerol, immediately mixed with 5 ml 5% TCA and allowed to stand on ice for 20 min. The samples were washed twice with distilled water. Lipids were extracted and analyzed as described above for the mass spectrometry analysis of lipids.

## Data availability

All the data produced for this work are contained within the article.

## Conflict of interest

The authors declare that they have no conflicts of interest with the contents of this article.
